# New Discovery of Calamitaceae from the Cisuralian in Northwest China: Morphological Evolution of Strobilus

**DOI:** 10.3390/biology13050347

**Published:** 2024-05-15

**Authors:** Xuelian Wang, Yunfa Miao, Yanzhao Ji, Bainian Sun

**Affiliations:** 1 Key Laboratory of Ecological Safety and Sustainable Development in Arid Lands/Key Laboratory of Desert and Desertification, Northwest Institute of Eco-Environment and Resources, Chinese Academy of Sciences, Lanzhou 730000, China; wangxuelian@nieer.ac.cn; 2 Hebei Research Center for Geoanalysis, Baoding 071000, China; jiyanzhao2024@163.com; 3 School of Earth Sciences, Lanzhou University, Lanzhou 730000, China; bnsun@lzu.edu.cn

**Keywords:** Hexi Corridor, Cisuralian, Calamitaceae, strobili, evolution

## Abstract

**Simple Summary:**

In this study, we provide descriptions of three fossil species, *Calamites cistii*, *Macrostachya* sp., and *Punctatisporites punctatus*, discovered in the Cisuralian of the eastern Hexi Corridor, northwestern China. Our findings offer insights into the evolution of Calamitaceae strobili during the Late Paleozoic.

**Abstract:**

Calamitaceae was the predominant arborescent element of the Late Paleozoic plant assemblages. However, there is currently controversy surrounding the evolutionary relationships of their four reproductive organs, and there is no unified understanding of the geographical distribution and species richness characteristics. This paper is based on the detailed description of the macro- and microstructures of *Calamites* and *Macrostachya* discovered in the Shanxi Formation of the Cisuralian in the eastern Hexi Corridor of northwestern China, and it discusses the evolutionary patterns of calamitean reproductive organs. The results indicate that the current specimens can be identified as the *Calamites cistii* and *Macrostachya* sp., and the in situ spores should exhibit distinct trilete marks, indicating that they belong to the *Punctatisporites punctatus*. The abundant fossil records suggest that the “*Calamostachys*” type should be considered as the ancestral type of strobili. Additionally, Calamitaceae was widely distributed in mid to low latitudes globally from the Pennsylvanian to the Cisuralian and coupled with paleogeographic distribution characteristics.

## 1. Introduction

Calamitaceae primarily represents arborescent plants within the Equisetales order, typically preserved as medullosan stems or near-cylindrical strobili fossils [1,2,3,4,5,6,7,8,9,10,11,12]. These plants first appeared in the late Devonian period and are a type of plant that grow rapidly, resembling bamboo, in the peat swamp ecosystems of lowland equatorial regions [13,14]. Throughout evolution and up to the present day, *Equisetum*, the living fossil herbaceous spore plant, remains the sole remnant molecular representation of this plant type [15]. The previously discovered Calamitaceae fossil specimens are mostly fragmented, typically preserving only partial organs of the dispersed plants. Our understanding of Calamitaceae mainly relies on the medullary casts of hollow stems or coalified imprints of branches, leaves, or strobili [7,8,9]. Faced with the gradually drying climate and exceptionally dynamic environment during the Late Paleozoic period, as well as the emergence of seed plants, the evolution process of Calamitaceae also underwent changes during this period.

Based on the current reliable fossil records, Calamitaceae was widely distributed in Europe, North America, and East Asia from the Late Paleozoic to the Mesozoic era. Among them, genera representing stem and foliage fossils include the following: *Archaeocalamites*, *Mesocalamites*, *Calamites*, *Paracalamites*, *Neocalamites*, *Gansuphyllites*, *Asterophyllites*, and *Annularia*, and the calamitean strobili mainly include four types, *Calamostachys*, *Palaeostachya*, *Macrostachya,* and *Cingularia* [1,2,3,4,5,14,16,17,18,19], of which *Palaeostachya* was founded by Weiss in 1876 and considered to represent the cone of Calamitaceae in the Late Paleozoic era. However, not all leaf fossil genera have corresponding strobilus types, and due to the limitations of fossil records, there is still some controversy regarding the evolutionary relationships of strobili [1,19,20,21].

In this paper, we report on two well-preserved species of *Calamites* and *Macrostachya* found in the Cisuralian Shanxi Formation of Yongchang, Gansu Province, in Northwest China. The intricate microstructure of the stem and the characteristics of in situ spores within the strobilus were thoroughly investigated. Additionally, this study delved into the evolutionary relationships of calamitean strobili.

## 2. Materials and Methods

**Figure 1:**
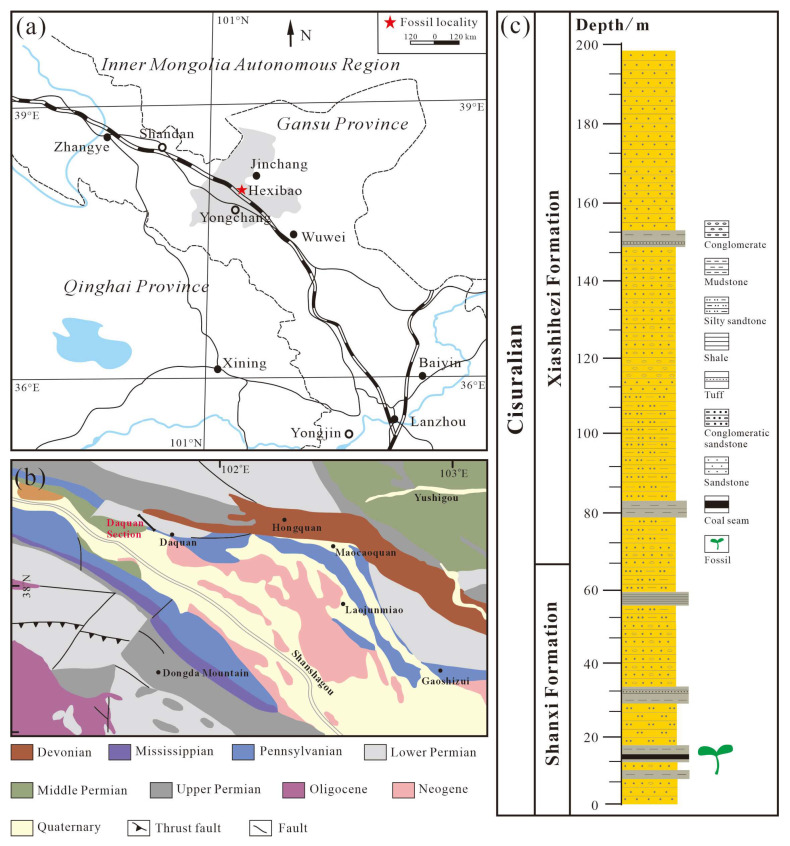
(**a**,**b**) Fossil locality and (**c**) stratigraphic column of the Cisuralian Shanxi Formation in the Yongchang Section, showing the fossil-bearing strata.

The research area belongs to the Longshou Mountain region, which is about 10 km north-west of Hexibao Town, Yongchang County (38°28′33″ N, 102°101′32″ E) [22]. This area exposes a well-preserved section of the Permian continental strata, named the Daquan section. All fossil specimens were collected from the lower part of layer 3 and layer 5 of the Shanxi Formation in Yongchang, Gansu Province, Northwest China. The stratigraphy of the Cisuralian Shanxi Formation has been discussed in detail in earlier work and comprises seven layers (Figure 1) [23].

The lower Shanxi Formation is considered to be the Cisuralian [22,24,25,26,27]. Palaeophytogeographically, the Cisuralian flora in the study area belongs to the possible boundary between the Cathaysian and Angaran floras [28]. Considering the occurrence of specific biomarkers of Cathaysia flora such as *Lepidodendron*, *Cathaysiodendron*, *Lobatannularia,* and *Tingia* in the Shanxi Formation, and the plant fossil and the palynomorph assemblage in the Yongchang area; the geological age in this area should be classified as the Artinskian-Kungurian [23].

The specimens were first photographed using a digital camera. Then, their morphological features were observed and photographed using a Leica MZ12.5 Stereo Microscope. The epidermis of the specimen stem was obtained using a method involving the Aqua regia and NaClO maceration, and the in situ spores were obtained by the maceration of the pre-treated strobilus in NaClO—Schulze’s solution [26]. The slides were then prepared and observed and photographed under the light microscope (LM) and scanning electron microscope (SEM). All fossil specimens are stored at the Institute of Palaeontology and Stratigraphy, Lanzhou University, located in Gansu Province, China.

## 3. Results

**Class:** Sphenopsida.

**Order:** Equisetales.

**Family:** Calamitaceae.

**Genus:***Calamites* Suckow, 1828.

**Species:***Calamites cistii* Brongniart.

**Figure 2:**
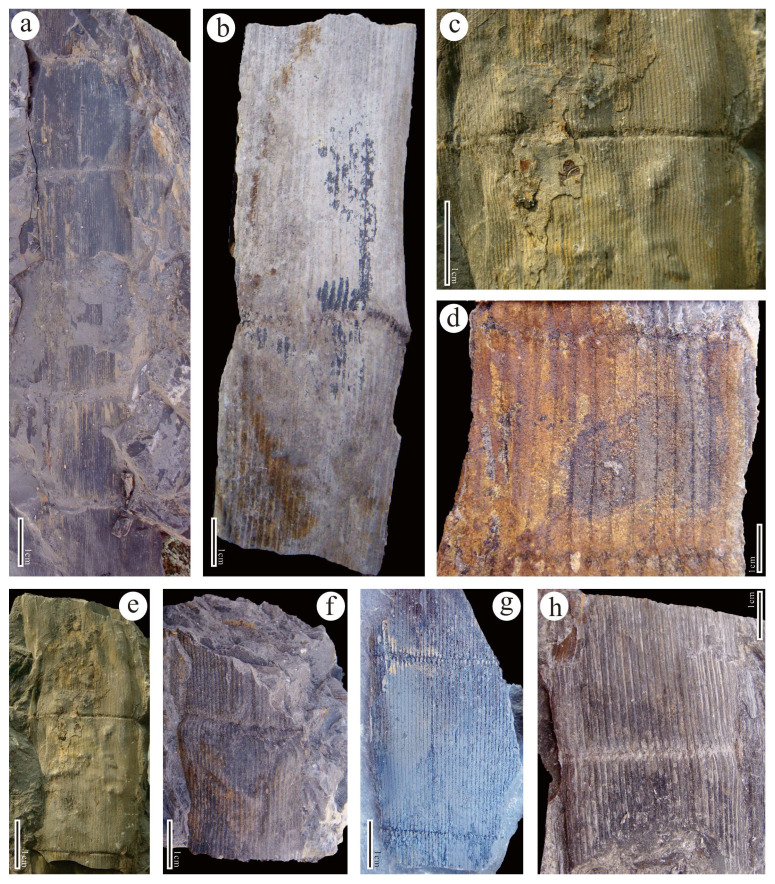
Morphological characteristics of *Calamites cistii* from the Cisuralian of Yongchang, Gansu. (**a**,**b**). Specimen no.: GSYC-LDGSW-2014-098; (**c**,**e**). Specimen no.: GSYC-LDGSW-2014-088; (**d**,**g**). Specimen no.: GSYC-LDGSW-2014-090; (**f**). Specimen no.: GSYC-LDGSW-2014-167; (**h**). Specimen no.: GSYC-LDGSW-2014-146. Scale bars = 1 cm.

**Specimens:** GSYC-LDGSW-2016-098 (A, B) (Figure 2a,b); GSYC-LDGSW-2016-088 (Figure 2c,e); GSYC-LDGSW-2016-090 (Figure 2d,g); GSYC-LDGSW-2016-167 (Figure 2f); GSYC-LDGSW-2016-146 (Figure 2h).

**Locality:** Yongchang County, Gansu Province, Northwest China.

**Stratigraphic horizon:** The Shanxi Formation, Cisuralian.

**Repository:** The fossil repository of the Institute of Palaeontology and Stratigraphy, Lanzhou University, Gansu Province, China.

**Figure 3:**
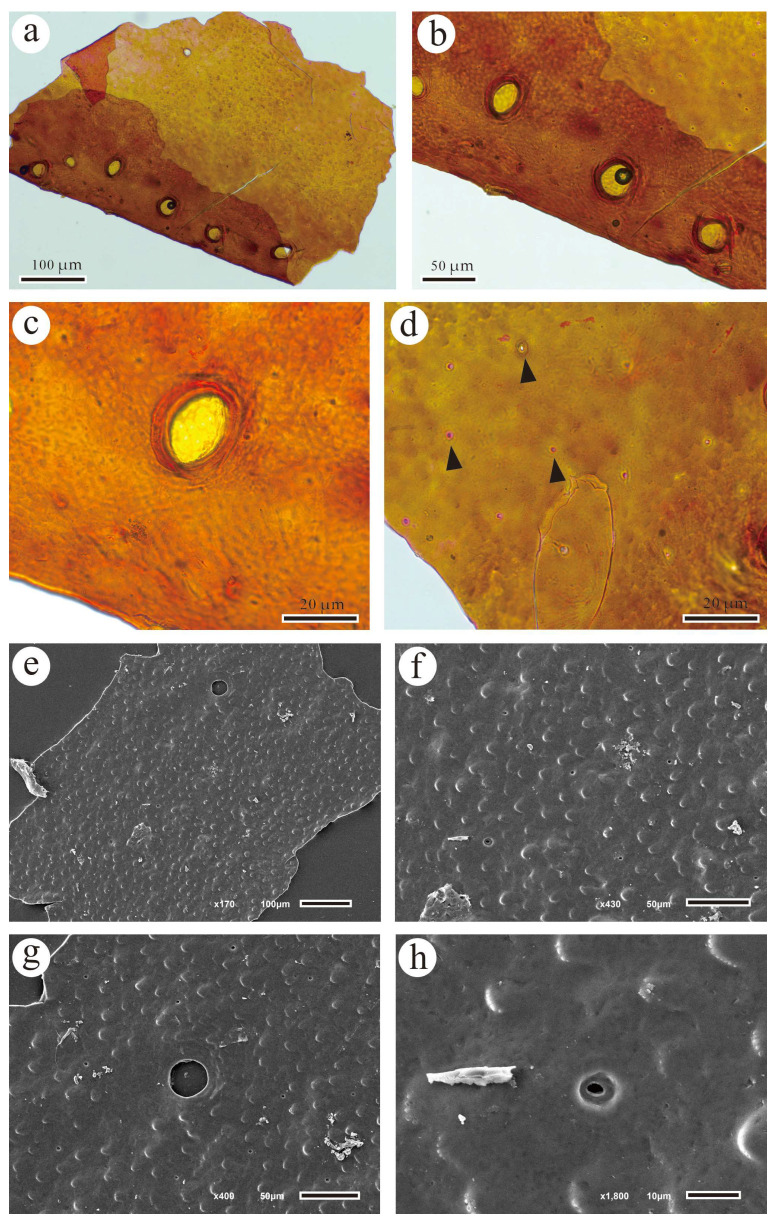
Micromorphology of Calamites cistii from the Cisuralian of Yongchang, Gansu. (**a**–**d**). Cuticles by light microscopy showing scars and circular pores (black arrows); (**e**–**h**). Cuticle by SEM. Scale bars = 100 μm (**a**,**e**), 50 μm (**b**,**f**,**g**), 20 μm (**c**,**d**), 10 μm (**h**).

**Description:** The current specimens have a node length of approximately 4 cm and a width of about 2.5 cm, with a length greater than the width (Figure 2a,h). The longitudinal ribs are flat and narrow, arranged alternately on the nodes, tapering gradually at both ends (Figure 2d). At the top of each rib, there is an oval-shaped leaf scar, with no clear scars observed on the nodes, and the longitudinal grooves are single-lined (Figure 2d). The stem epidermis is relatively thick, with no stomata observed, and the leaf scars are oval-shaped (Figure 3a,c), with thickened outer edges (Figure 3c,g). Additionally, numerous circular pores are evenly distributed on the surface of the epidermis (Figure 3d–f), with the centers of the pores depressed (Figure 3h).

**Remarks:** The current specimen exhibits the main characteristics of the *Calamites*, and its described morphology is consistent with the *Calamites cistii*. It can be confirmed that this species is a new discovery of the Cathaysian flora.

**Genus:***Macrostachya* Schimper, 1869.

**Species:***Macrostachya* sp.

**Figure 4:**
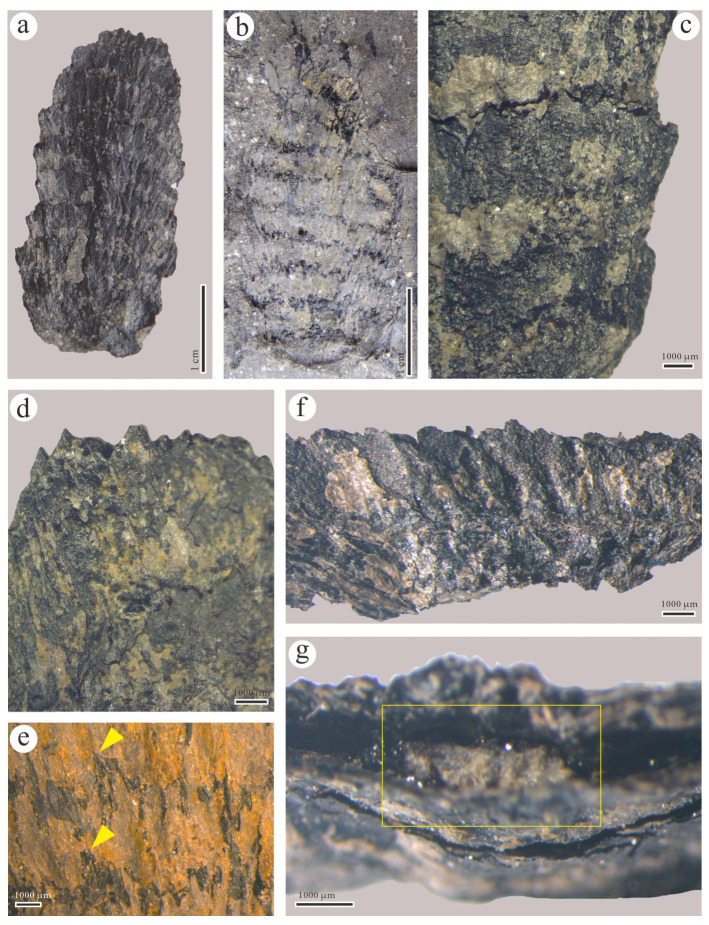
Morphological characteristics of *Macrostachya* sp. from the Cisuralian of Yongchang, Gansu. (**a**,**b**). Specimen no.: GSYC-LDGSW-2014-003; (**c**–**f**). Sporophylls (yellow arrows); g. Strobilus axis (yellow box). Scale bars = 1 cm (**a**,**b**), 1000 μm (**c**–**g**).

**Specimens:** GSYC-LDGSW-2016-003 (A, B) (Figure 4).

**Locality:** Yongchang County, Gansu Province, Northwest China.

**Stratigraphic horizon:** The Shanxi Formation, Cisuralian.

**Repository:** The fossil repository of the Institute of Palaeontology and Stratigraphy, Lanzhou University, Gansu Province, China.

**Figure 5:**
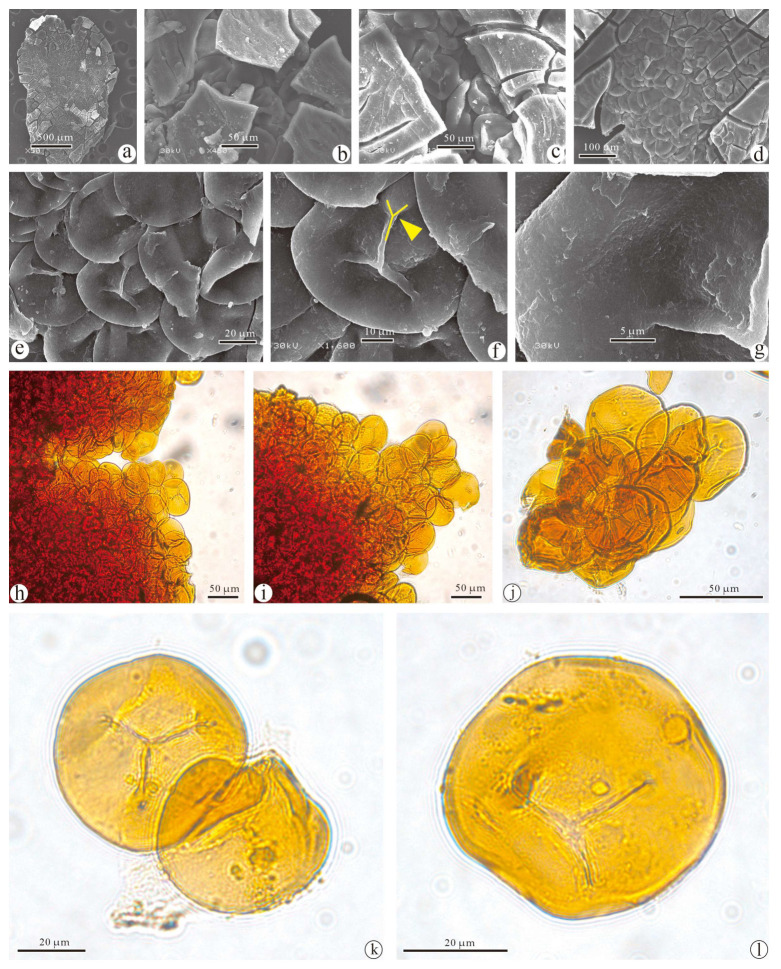
In situ spore characteristics of *Punctatisporites punctatus* by LM and SEM. (**a**). Sporophyll by SEM; (**b**–**g**). In situ spores by SEM, “Y” shape at the end of trilete mark in (**f**) (yellow arrow); (**h**–**l**). In situ spores by LM. Scale bars = 500 μm (**a**), 100 μm (**d**), 50 μm (**b**,**c**,**h**–**j**), 20 μm (**e**,**k**,**l**), 10 μm (**f**), 5 μm (**g**).

**Description:** The current specimen is well preserved (Figure 4a,b). The strobilus is cylindrical and relatively small, with a central portion about 3 cm long and 1.3 cm wide (the top diameter is approximately 0.94 cm and the bottom diameter is approximately 0.42 cm). Each round of sporophylls is arranged in a shingled pattern with tight interconnections between them, and their bases are connected together and arranged parallelly (Figure 4c). A whorl composed of 32 sporophylls appears in each nodal area, alternating from the previous node to the next one, arranged parallelly and extending to about one-third of the previous round of sporophylls (Figure 4f). The apex of the sporophyll is upright and relatively pointed (Figure 4d), with an average diameter of 0.7 mm, widening to 1.5 mm as the axis develops, and then tapering near the apex and becoming less than 0.7 mm (Figure 4e, yellow arrows). The axis of the specimen is nodally arranged, with a diameter of about 0.15 mm at the nodes (Figure 4g, yellow box). The attachment of sporangiophores is unclear, but based on the scanning of a complete sporophyll under an electron microscope (Figure 5a), a large number of spores are found beneath each sporophyll, indicating that each sporophyll corresponds to at least one sporangium. The fine structure characteristics of the sporophylls in the current specimen are not identified, but detailed features of the in situ spores are obtained.

**Remarks:** The current specimen does not provide information about the attachment state of sporangiophores, but its clear arrangement of bracts suggests its classification into the *Macrostachya* is appropriate.

**Genus:***Punctatisporites* Potonie & Kremp, 1954.

**Species:** Punctatisporites punctatus Ibrahim.

**Specimens:** GSYC-LDGSW-2016-003 (Figure 5).

**Locality:** Yongchang County, Gansu Province, Northwest China.

**Stratigraphic horizon:** The Shanxi Formation, Cisuralian.

**Repository:** The fossil repository of the Institute of Palaeontology and Stratigraphy, Lanzhou University, Gansu Province, China.

**Description:** The in situ spores are oval-shaped, with a diameter of about 50–60 μm (Figure 5a,j). The spore surface is smooth and exhibits a trilete mark (Figure 5k,l). The ends of the trilete mark form a “Y” shape (Figure 5f, yellow arrow). Based on the features of the in situ spores, the spore type of the current specimen belongs to the genus *Punctatisporites* [29]. The in situ spores of the current specimen show no differentiation; only one type is currently present.

**Remarks:** In situ spores are of the *Punctatisporites* and the same as those described from *Punctatisporites punctatus*.

## 4. Discussion

### 4.1. Comparisons

#### 4.1.1. Comparison of *Calamites cistii* with Fossil Taxa

The stems of the nutritional organs of the Calamitaceae plants exhibit a characteristic feature of “internode”. In the absence of preserved leaves, distinguishing the systematic classification position of specimens relies solely on the characteristics of the segments on the stems. *Archaeocalamites*, *Mesocalamites,* and *Neocalamites* are distinguished from the current specimens by their extremely short internode distances. *Paracalamites*, *Asterophyllites,* and *Gansuphyllites* are differentiated from the current specimens by their ribs passing straight through the nodes without crossing. Fossil species in the *Annularia* currently only present leaf forms, with no discoveries regarding stems [13,30]. Therefore, the current specimens are classified as *Calamites* due to their wide and intersecting distribution of segments.

The internodal lengths of the current specimens are greater than their widths, and the lower leaf scars are elliptical in shape. These features distinguish it from the *Calamites cruciatus*, *C*. *suckowii*, and *C*. *undulatus* [13]. While the *C*. *taiyuanensis* shares similarities with the current specimens, they have a larger length-to-width ratio and longitudinal ribs. *Calamites cistii* has a similar length-to-width ratio and features other similarities to the current specimens. Therefore, identifying the current specimens as *Calamites cistii* is appropriate.

#### 4.1.2. Comparison of *Macrostachya* sp. with Fossil Taxa

There are several genera representing the calamitean strobili, including *Asterocalamites*, *Protocalamostachys*, *Calamostachys*, *Palaeostachya*, *Cingularia*, *Mazostachys*, *Macrostachya*, and *Metacalamostachys*. The most common and recognized genera are the *Calamostachys*, *Palaeostachya*, and *Macrostachya*, which are distributed between the late Devonian and the Permian eras. The distinguishing features of these reproductive organs are mainly based on the attachment mode of the sporangiophore [5,31,32]. The sporangiophore of *Calamostachys* is attached at right angles to the axis, in the middle of the internode. The *Palaeostachya* has the sporangiophore attached in the axil of the sporophylls, while specimens with characteristics of calamitean strobilus but with an uncertain attachment mode of the sporangiophore are classified under the *Macrostachya*.

The current specimen is well preserved, and through the identification of the in situ spore types, it is confirmed to be a strobilus of Calamitaceae. However, the attachment of the sporangiophore inside the strobilus could not be determined. Therefore, it is classified under the *Macrostachya*. Four species of the *Macrostachya* are currently known (Table 1). Among them, the *M*. *conica*, *M*. *thompsonii*, and *M*. *infundibuliformis* have elongated strobili distinct from the current specimen, and the *M*. *thompsonii* differs from other species within the genus due to its heterosporous nature. The morphology of *M*. *huttoniaeformis* is very similar to that of the current specimen, although they differ slightly in size, making it difficult to distinguish between them. The sporophylls of the former are narrowly triangular, in contrast to the elliptical shape of the latter. However, the current specimen is heavily carbonized, and its morphology may have been affected by environmental factors, thus it is provisionally designated as *Macrostachya* sp.

#### 4.1.3. Comparison of Punctatisporites punctatus with Fossil Taxa

It is generally believed that spores producing trilete originate from fern-allie plants, and the affinity of these plants can only be identified from in situ spores [35,36]. Based on detailed studies, the comparison usually involves the *Cyclogranisporites*, *Verrucosisporites* and *Punctatisporites*, which are in situ spores with trilete characteristics from the Paleozoic, thus helping us to narrow the scope of comparison for this study. *Cyclogranisporites* was typically circular in shape, with a non-divergent trilete almost reaching the margin, ranging in size from 10–70 μm, and was commonly found in marattialean, zygopterid, and botryopterid ferns [29,35]. *Verrucosisporites* was circular to oval in shape with uneven trilete lengths, ranging in size from 20 to 140 μm, and was generally believed to belong to ferns and some noeggerathialeans [29,36]. *Punctatisporites* was circular to subcircular, ranging in size from 10 to 140 μm, with a trilete that was divergent and almost reaching the margin, and was also believed to originate from ferns and some noeggerathialeans [29,35,36]. While size cannot be used as the sole criterion for assessment, the characteristic of divergent trilete in the in situ spores of the current specimen justifies its classification as the *Punctatisporites* (Table 2).

The most distinctive feature of the *Punctatisporites* is its nearly circular shape with clearly visible, elongated trilete marks and a smooth surface, appearing brownish yellow. The in situ spore characteristics of the current specimen clearly indicate its attribution to this genus. The *Punctatisporites* encompasses a considerable number of species, with approximately over 80 identified [29]. The corresponding parent plants are unknown, with a wide range of possible types, commonly found among plants with nodes. The differences between species mainly lie in the morphology of the trilete marks: their length and whether their ends are bifurcated. In the current specimen, the trilete marks on the in situ spores reach the equatorial edge and exhibit prominent bifurcation, forming a distinctive “Y” shape. After excluding the species of the *Punctatisporites* with non-bifurcated trilete marks and conducting a detailed comparison, the in situ spores of the current specimen are likely to belong to *Punctatisporites punctatus*. The detailed experimental analysis in this paper indicates that the in situ spores of the current specimen show no differentiation, suggesting that one of the parent plants of *Punctatisporites punctatus* is likely to be the *Macrostachya*, and the findings of the current study enrich the Late Paleozoic record of in situ spores.

### 4.2. The Strobilus Evolution of Calamitaceae

The most frequently occurring and widely distributed reproductive organ types among the Calamitaceae are *Calamostachys* and *Palaeostachya*, to the extent that early scholars often focused their discussions on the evolution of these types in relation to other reproductive organ types. Since Weiss [1] first proposed distinguishing between types primarily based on the attachment position of the sporangiophore, most scholars have made it clear that the connection between the sporangiophore and the internode is merely a simple junction and does not represent a connection between the internal vascular structures of the plant [1,14,16,17,21]. Therefore, scholars have proposed three evolutionary pathways for these types of calamitean strobili.

(1) Hickling and Weiss proposed that the *Palaeostachya* evolved from the *Calamostachys* through a process called “phyletic slide”. The sporangiophore of *Calamostachys*, initially positioned in the middle of the internode, underwent evolutionary changes over time, descending from the middle to the axil of the sporophylls, thus forming the *Palaeostachya*. Hickling provided evidence supporting this evolutionary trajectory, suggesting that the *Calamostachys* is more likely to be the ancestral type. As for the *Cingularia* and other rare types, they could also be considered ancestral groups, but there is no evidence supporting this possibility [20]. (2) Some scholars believe that the most primitive position of the sporangiophore in calamitean strobilus is in the axil of the sporophylls, followed by an upward evolutionary trajectory. According to this view, the sporangiophore of the fossil species representing the ancestral type, the *Palaeostachya decacnema*, later moved upward to the middle of the internode, representing the *Calamostachys*. Then, the sporangiophore descended again to the middle of the internode, represented by the *P*. *vera* and *P*. *andrewsii*. However, this evolutionary pathway cannot be proven, and it is suggested that the phenomena that cannot be explained may be the most correct [21]. (3) The proposal of the third evolutionary pathway is closely related to the appearance of the *Mazostachys*. The position of the sporangiophore in the *Mazostachys* is located at the top of the internode, below the back of the sporophylls, which is the same as in the *Cingularia*. Therefore, later, these two genera were considered to belong to the same type. Kosanke [19] believed that the sporangiophore located in the axil of the sporophylls is a derived type, meaning that the *Palaeostachya* is definitely not the ancestral type. He also agreed with Hickling’s view that the *Calamostachys* is the ancestral type, but this genus should evolve in two directions: the sporangiophore moving downward evolves into the *Palaeostachya*, while simultaneously, the sporangiophore could also move upward to evolve into the *Mazostachys* or the *Cingularia* [19]. Types with unknown sporangiophore positions were assigned to the *Macrostachya* [1,3,14,17,18].

Furthermore, the earliest strobilus records also originate from the late Devonian period in Hunan, South China, named the *Calamostachys hunanensis*, characterized by sporangiophore attachment at the nodes [37]. In the early Carboniferous era, European calamitean strobilus types differ significantly from those in Chinese specimens, characterized by sporangiophore attachment at the axils of sporophylls, with the *Palaeostachya ettingshauseni* being a typical representative [2]. Subsequently, during the late Carboniferous period, *Palaeostachya* became widespread in European and North American plant communities, with numerous discoveries and diverse strobilus types in Northern China. *Mazostachys*, with sporangiophore attached to the top of the nodes, and *Macrostachya*, with uncertain attachment positions, also appeared. However, by the Permian era, calamitean reproductive organ types became monotonous, with only the *Palaeostachya* and *Macrostachya* distributed in China [33,34,37,38,39], with only two undetermined species of the *Palaeostachya* recorded in Guangdong, China [40]. The distribution characteristics of the Calamitaceae during the geological period are consistent with the earliest reported fossil records. Based on this, it is speculated in this paper that they are mainly distributed in mid–low latitude regions and underwent an evolution in their foliage and strobilus types during this process. The paleogeographic distribution characteristics of Calamitaceae also indicate that *Calamostachys* appeared earlier than other strobilus types, providing reliable evidence for the view that the “*Calamostachys*” type is ancestral [19].

**Figure 6:**
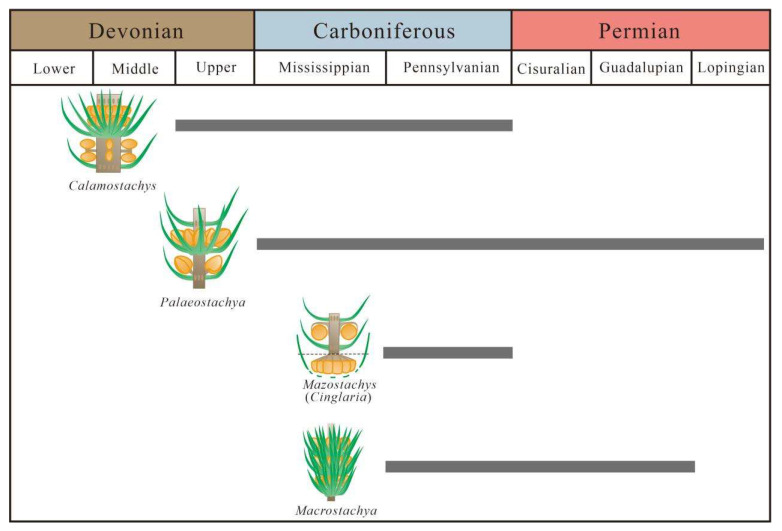
The age distribution and evolution of calamitean strobili.

After over half a century of development in paleobotany, there have been numerous discoveries of calamitean strobilus fossils in the Late Paleozoic period worldwide. Based on the distribution evidence of reproductive organs (Figure 6), it is evident that the *Calamostachys* appeared earliest in the late Devonian era, which preceded other types. Therefore, there is a strong possibility that the “*Calamostachys*” type is the ancestral genus. Meanwhile, the *Palaeostachya* and *Mazostachys* appeared during very close geological periods, and other types of strobili only emerged in the late Carboniferous era. Therefore, the authors of this study have reason to believe and support the evolutionary pathway proposed by Kosanke [19] as more reasonable.

## 5. Conclusions

(1) This paper reports two calamitean species from the Shanxi Formation of the Cisuralian in Yongchang, Gansu: *Calamites cistii*, *Macrostachya* sp., with in situ spores of the *Punctatisporites punctatus*. A detailed systematic description and comparative discussion of their macro- and microstructures characteristics are provided.
(2) Based on detailed fossil records, there is a higher possibility that the *Calamostachys* represents the ancestral type of calamitean strobili. The initial attachment position of the sporangiophore should be located in the middle of the internode, from which other types of strobili morphology could have evolved, coupled with the reliable geological records of the Calamitaceae strobili.

## Figures and Tables

**Table 1:** Morphological comparisons of *Macrostachya* fossils.

Species	Strobilus		Sporophyll					Axis (cm)	Reference
	Shape	Bracts Connection	Shape	Apex	Angle/ Attachment	No. of Bracts per Whorl	Whorls		
*Macrostachya* sp.	Cylindrical	Whorled, imbricate	Ovoid	Pointed	>45°	30–32	10	0.7	Present study
*M. conica*	Long- tubular	Whorled, imbricate	Taper	Pointing triangular	/	30–40	/	0.5	[33]
*M. huttoniaeformis*	Cylindrical	Whorled	Long triangle	Pointed	/	50–60	/	/	[14]
*M. thompsonii*	Long- tubular	Whorled, imbricate	Fanshaped	Cuspidate	<45°	32–36	50–53	/	[34]
*M*. *infundibuliformis*	Long- tubular	Whorled	Elongate	Pointed	/	30–40	/	0.20	[34]

**Table 2:** Comparison between *Punctatisporites* and similar genera.

Genus	*Punctatisporites*	*Cyclogranisporites*	*Verrucosisporites*
Morphology	circular to subcircular	circular	circular to oval
Trilete	Bifurcate and almost reaching the margin	Non-bifurcate and reach the margin	Uneven lengths
Diameter (μm)	10–140	10–70	20–140
Parent plants	Ferns and noeggerathialeans	Ferns	Ferns and noeggerathialeans
Reference	[29,35,36]	[29,35]	[29,36]

## Data Availability

All data dealing with this study are reported in the paper.
